# Lipid metabolism of leukocytes in the unstimulated and activated states

**DOI:** 10.1007/s00216-020-02460-8

**Published:** 2020-02-14

**Authors:** Juan Carlos Alarcon-Barrera, Johannes H. von Hegedus, Hilde Brouwers, Evelyne Steenvoorden, Andreea Ioan-Facsinay, Oleg A. Mayboroda, Alejandro Ondo-Mendez, Martin Giera

**Affiliations:** 1 Center for Proteomics and Metabolomics, Leiden University Medical Center (LUMC), Albinusdreef 2, 2333 ZA Leiden, The Netherlands; 2 Escuela de Medicina y Ciencias de la Salud, Universidad del Rosario, Bogotá, 111221 Colombia; 3 Department of Rheumatology, Leiden University Medical Center (LUMC), 2300 RC Leiden, The Netherlands

**Keywords:** Lipidomics, CD4+, CD14+, Neutrophils, Lipidyzer™, IPA extraction

## Abstract

Lipidomics has emerged as a powerful technique to study cellular lipid metabolism. As the lipidome contains numerous isomeric and isobaric species resulting in a significant overlap between different lipid classes, cutting-edge analytical technology is necessary for a comprehensive analysis of lipid metabolism. Just recently, differential mobility spectrometry (DMS) has evolved as such a technology, helping to overcome several analytical challenges. We here set out to apply DMS and the Lipidyzer™ platform to obtain a comprehensive overview of leukocyte-related lipid metabolism in the resting and activated states. First, we tested the linearity and repeatability of the platform by using HL60 cells. We obtained good linearities for most of the thirteen analyzed lipid classes (correlation coefficient > 0.95), and good repeatability (%CV < 15). By comparing the lipidome of neutrophils (PMNs), monocytes (CD14+), and lymphocytes (CD4+), we shed light on leukocyte-specific lipid patterns as well as lipidomic changes occurring through differential stimulation. For example, at the resting state, PMNs proved to contain higher amounts of triacylglycerides compared to CD4+ and CD14+ cells. On the other hand, CD4+ and CD14+ cells contained higher levels of phospholipids and ceramides. Upon stimulation, diacylglycerides, hexosylceramides, phosphatidylcholines, phosphoethanolamines, and lysophosphoethanolamines were upregulated in CD4+ cells and PMNs, whereas CD14+ cells did not show significant changes. By exploring the fatty acid content of the significantly upregulated lipid classes, we mainly found increased concentrations of very long and polyunsaturated fatty acids. Our results indicate the usefulness of the Lipidyzer™ platform for studying cellular lipid metabolism. Its application allowed us to explore the lipidome of leukocytes.

**Electronic supplementary material:**

The online version of this article (10.1007/s00216-020-02460-8) contains supplementary material, which is available to authorized users.

**Figure:**
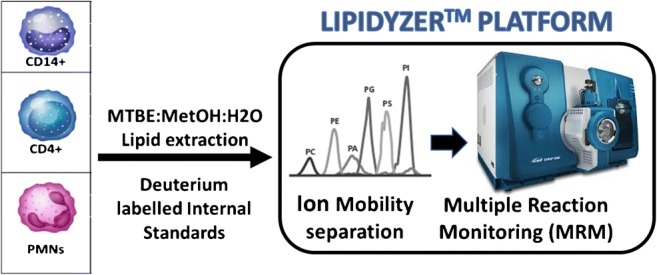
Graphical abstract

## Introduction

Lipids are a dynamic group of molecules consisting of different classes and subclasses [1]. The biological functions of lipids are very diverse, ranging from their role as structural elements of cellular membranes, and energy storage pool, to the regulation of intracellular signaling [2, 3]. Lipidomics has emerged as a useful strategy to measure and study lipids and lipid-mediated cellular processes [4, 5]. Traditionally, shotgun or multi-dimensional mass spectrometry-based lipidomics approaches have been used [6]*.* However, the lipidome contains numerous isomeric and isobaric species resulting in a significant overlap between different lipid classes. On this regard, differential mobility spectrometry (DMS) has just recently evolved as a useful tool to overcome this issue [7, 8]. In DMS, the ions formed by electrospray ionization are guided through a gas stream between two planar electrodes in which gas phase separation on the millisecond scale can be achieved by fine tuning separation and compensation voltages in combination with organic modifiers affecting the molecular shape [8]. For lipidomics analysis, this technology has been an important development as it allows the gas-phase separation of lipid classes thereby overcoming isobaric cross-talk [9]. Recently, a commercial platform, called the Lipidyzer™, has become available as a validated quantitative lipidomics platform. The Lipidyzer™ quantifies roughly 1.100 lipid species across 13 lipid classes employing a series of deuterium-labeled internal standards. The platform comprises a flow injection system coupled to a QTrap® 5500 operated in electrospray ionization mode (ESI), equipped with a SelexION DMS device. By applying a compensation voltage (COV) within the DMS, cell lipid classes are separated in the gas phase before entering the QTrap® mass spectrometer (MS) thereby overcoming isobaric overlap between the classes. Subsequently, the MS is operated in multiple reaction monitoring (MRM) mode, selectively scanning the individual (separated) lipid classes (8) (cf materials and methods and Electronic Supplementary Material (ESM) S1 and S2). Thus, far few reports have evaluated the platform; Contrepois et al. tested the Lipidyzer™ and compared its performance with that of conventional LC-MS-based untargeted lipidomics strategies. The authors concluded that the Lipidyzer™ is a robust platform delivering comparable results with conventional lipidomics analysis strategies [10]. Just recently, Cao et al. described a high reproducibility of the platform when analyzing tissue samples [11]. However, while the platform has shown excellent results for reproducibility, relative quantification, and fatty acid compositional analysis [12], it has some limitations. While lipid species are being characterized including fatty acid carbon and double bond number, double bond position and geometry are not defined. Technologies helping to possibly overcome such limitations involve photochemical derivatization [13] and ozonolysis [14]. Moreover, care should be taken when reporting absolute quantifications as no report has yet described the actual accuracy of the platform. Nevertheless, the Lipidyzer™ platform together with several other technological developments has made lipidomics analysis applicable to the study of lipid metabolism and biology. For many years, studies about the effect of dietary lipids on health and disease were mainly focused on cardiovascular diseases. However, it has become accepted that lipid metabolism is linked to the development of different pathologies as for example diabetes, blood clotting, Alzheimer’s disease, and cancer, as well as in physiological processes like the inflammatory response [15, 16]. The biosynthesis of red blood cells, monocytes, polymorphonuclear neutrophils (PMNs), and platelets has been shown to be largely dependent on lipids as energy source and modulators of hematopoietic stem and progenitor cell (HSPC) differentiation [12].

Immune cells derived from both myeloid and lymphoid lineages work together in immune and inflammatory response, preventing and limiting infection and promoting tissue regeneration and homeostasis [17]. However, to date, no comprehensive study has described the lipidome of these lineages in resting and activated states. We here set out to apply cutting-edge technology and obtain a comprehensive lipidomics overview of PMNs, monocytes (CD14+), and the major population of circulating lymphocytes (CD4+). Additionally, we compared their lipidomes and shed light on lipidomic changes that occur upon stimulation. We initially investigated the platforms’ linearity and repeatability using different amounts of HL60 cells as readily available surrogate cells. Subsequently, we applied the Lipidyzer™ platform to generate quantitative lipid profiles of PMN, CD14+, and CD4+ cells in the resting and activated states. Our data forms the basis for a better understanding of immune cell-specific lipid metabolism and is a rich source comprehensively describing immune cell-specific lipidomes and lipidomic changes during activation.

## Materials and methods

### Cell culturing

Human leukemia cells (HL60) were obtained from the ATCC (Manassas, VA) and were grown in a humidified atmosphere of 5% CO_2_ at 37 °C and maintained in RPMI 1640 from Gibco (Carlsbad, CA) supplemented with 10% heat-inactivated fetal bovine serum from Hyclone (Logan, UT) and 1X antibiotic mix (Gibco, Carlsbad, CA). Cell density was kept between 0.2 and 2 × 10^6^ cells/mL.

### Isolation of PMNs, CD14+ monocytes, and CD4+ T cells

This study was approved by the local medical ethical committee of the LUMC (METC), and written informed consent was given by all donors. Isolation of human PMNs from three fresh 50-mL EDTA blood containers was done via DextranT500 sedimentation (Pharmacosmos, Holbaek, Denmark), taking only the upper layer followed by Ficoll density gradient separation. Hypotonic lysis removed the remainder of erythrocytes. Purity was checked by FACS (LSRIII, BD, San Jose, USA), staining the cells with 2 CD3-AF700 (clone UCHT1)/CD15-APC (clone HI98)/CD16-PE (clone 3G8)/CD19-FITC (clone HIB19). Isolated PMNs were resuspended in Dulbecco’s phosphate-buffered saline with MgCl_2_ and CaCl_2_ (Merck, Darmstadt, Germany). Cell purity was > 98%.

Human peripheral blood mononuclear cells (PBMCs) were isolated by Ficoll density gradient from three healthy 50 mL donor buffy coats (Sanquin, Amsterdam, The Netherlands). Blood monocytes were isolated by positive selection from PBMCs using MACS CD14 Microbeads (Miltenyi Biotec, Cologne, Germany), and purity was checked by FACS (LSRIII, BD, San Jose, USA) staining the cells with CD14-PE (clone MφP9). Cell purity was > 98%.

CD4+ T cells were obtained by further purifying the CD14 negative fraction with Dynabeads™ FlowComp Human CD4 positive Isolation Kit (Invitrogen) according to the manufacturer’s protocol. CD4+ T cells were kept in IMDM medium (Gibco) containing 4500 mg/L d-glucose and l-glucose, 10% FCS (LUMC apothecary), 100 U/mL penicillin and streptomycin (LUMC apothecary), and 2 mM Glutamax (LUMC apothecary). Cell purity was > 97%.

### Cell stimulation

Both PMNs and CD14+ monocytes were stimulated for 10 min with 4 μM calcium ionophore A23178 (Merck, Darmstadt, Germany). CD4+ cells were activated using αCD3/CD28 beads (Gibco). Purity and activation were checked by FACS (LSRIII, BD, San Jose, USA) staining the cells with CD3-PE (clone SK7)/CD4-APC (clone SK3)/CD8-FITC (clone SK1)/CD14-PEcy7 (clone M5E2) and activation using CD25-AF700 (clone BC96). The percentage of CD25+ cells was > 35%.

### Lipid extraction

For quantitative lipidomics analysis using the Lipidyzer™ platform, the cells were washed twice with PBS (+/+) containing 0.1% fatty acid free BSA after stimulation. The pelleted cells were stored at − 80 °C until analysis. For Lipidyzer™ analysis, 100 μL IS (Lipidyzer™ internal standard kit, containing > 50 labeled internal standards for 13 lipid classes) in methanol:dichloromethane (50:50 (v/v)) and 250 μL of 2-propanol (IPA) was added to 5 × 10^6^ cells. After 30 min of agitation, the samples were centrifuged for 10 min at 13.200*g* at 20 °C. The supernatant was collected, and the pellet was subjected to an additional extraction using 200 μL of IPA. The samples were then dried under a gentle stream of nitrogen, and suspended in 250 μL 10 mM ammonium acetate in (50:50 (v/v)) methanol:dichloromethane.

### Lipid measurement

Acquisition and quantification was performed using the Lipidyzer™ platform, consisting of a QTrap 5500 mass spectrometer (Sciex) with DMS, coupled to a Shimadzu Nexera X2 LC system, for flow injection, and the Lipidomics workflow manager software [8]. A detailed description of the quantitation process can be found in references [10, 11]. Briefly, 50 μL of resuspended sample was injected twice using a Shimadzu SIL 30AC autosampler into a running buffer consisting of 10 mM ammonium acetate in 50:50 (v/v) dichloromethane:methanol at a flow rate of 7 μL/min. Two different methods were applied. Method #1 operated with active DMS separation under the following conditions, DMS temperature low, modifier (propanol) composition low, separation voltage 3500 V, DMS resolution enhancement low. The DMS cell was not activated for method #2. The MS operated under the following conditions: curtain gas 17, CAD gas medium, ion spray voltage 4100 V in ESI+ mode and − 2500 V in ESI− mode, temperature 200 °C, nebulizing gas 17, and heater gas 25. First, PC, PE, LPC, LPE, and SM lipid classes were analyzed according to the method detailed in ESM S1. Next, FFA, TAG, DAG, CER, DCER, LCER, HCER, and CE lipids were analyzed applying method #2 as outlined in ESM S2. Each MRM Q1/Q3 pair is specific to a lipid species where Q1 is the precursor mass and Q3 is the mass of one of its constituent fatty acid product ions. Quantification of lipid species was achieved by internal calibration using several deuterated internal standards (IS) (Sciex cat# 504156) (cf ESM S1 and S2) for each lipid class within the lipidomics workflow manager. Each lipid species is corrected by the closest deuterated IS within its lipid class, in terms of carbon and double bond number of the fatty acid side chain. Subsequently, the obtained area ratio is multiplied by the concentration of the respective IS and corrected for volume and weight; as a consequence, this quantitation can be considered accurate within a specified quantitative bias. The so-obtained lipid concentration is then converted to nmol/50 × 10^6^ cells, taking into account that 5 × 10^6^ cells have been used in our experiments. On every day of analysis, the system was initially cleaned and calibrated. For additional details, please also refer to reference [10].

The following 13 lipid classes were quantitatively assessed: CE, cholesterol ester; CER, ceramides; DAG, diacylglycerides; DCER, dihydroceramides; FFA, free fatty acids; HCER, hexosylceramides; LPC, lysophosphatidylcholine; LPE, lysophosphatidylethanolamine; PC, phosphatidylcholine; PE, phosphatidylethanolamine; SM, sphingomyelin; and TAG, triacyltriglycerides. Although this nomenclature slightly deviates from the recommendations of LipidMaps, we adapted the given abbreviations as it facilitates a direct comparison between the raw data from the Lipidyzer™ platform and the results presented in the manuscript.

### Expression of results and statistical analysis

**Fig. 1:**
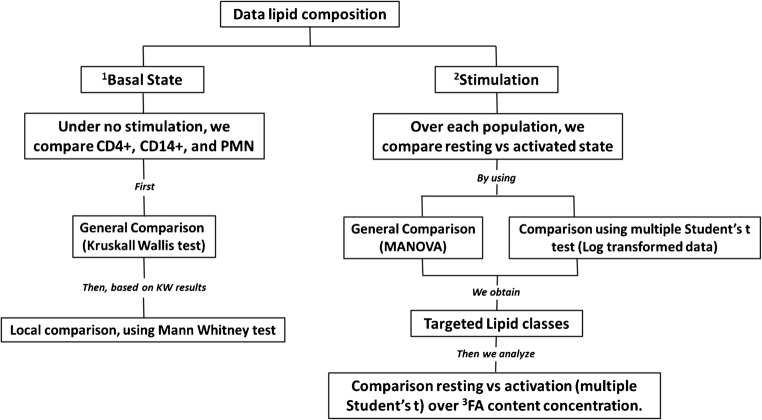
Data processing diagram. The following flowchart is a schematic guideline of how the data was processed for this study. The data was divided in two sections: unstimulated state and stimulation. ^1^For the unstimulated state, all cell populations were compared. ^2^For the stimulation part, a cell-specific comparison between resting and activation was carried out. ^3^In this sub-section, we explore the FA composition for specific lipid classes selected from the analysis of the stimulation part. Data was tested using nonparametric tests for raw data or parametric tests upon log transformation. Data was mainly lipid fractional composition except for FA content for which we used lipid concentration as observed changes were better visible

The Lipidyzer™ platform provides several readouts which are as follows: lipid class concentration (nmol/50 × 10^6^ cells), lipid species concentration (nmol/50 × 10^6^ cells), and fatty acid concentration (nmol/50 × 10^6^ cells) being the concentration of all lipids of a specific lipid class containing a specific fatty acid species. Moreover, compositional data (%) for the aforementioned items is being presented. Please refer to the entire lipidomics datasets as presented in ESM S3 to S5 for further details. All data was normalized with cell numbers. All values were expressed as mean ± SD. Missing values were replaced with the lowest data measured, unless more than 50% of the data was missing, in which case the lipid was discarded. For statistical analysis, we used R 3.6.1 and Graphpad Prism 8. Parametric tests were performed (MANOVA, and Student’s *t*) for data with a normal distribution; otherwise, a nonparametric test was used (Kruskal-Wallis and Mann-Whitney *U*) (Fig. 1). The significance level was set at *p* < 0.05; the Holm-Sidak method was used to corroborate the significance.

## Results

### Analytical evaluation

The Lipidyzer™ platform has been validated for plasma using a dichloromethane modified Bligh and Dyer extraction of lipids [9]. However, the approach uses large amounts of chlorinated organic solvents and did not seem appropriate to our study using small amounts of purified immune cells. Many different lipid extraction methods exist, and most result in method-specific recoveries and lipid coverages [18]. As this study is not about a comparison of different extraction methods and deals with small amounts of immune cells, we opted for repeated IPA extraction, a solvent which has been found useful for lipidomics analysis [19]. Nevertheless, we investigated the linearity (cell numbers) and repeatability (replicates) of this extraction method using the human lymphoma cell line HL60 (Table 1). The observed correlation coefficient was in most cases higher than 0.9. Under our conditions, the abundance of DCER was low compared to that of the other lipid classes, and considerably close to the measured values in the blank (< 1.5× blank). For FFA, we observed some background signals averaging roughly 25 nmol/10^6^ cells for the entire lipid class. Hence, we assessed linearity up to 50 × 10^6^ cells and established a lower cut-off for the use of the platform at an empirical value of at least 1.5× blank or approximately 10 × 10^6^ cells being - 65.8 nmol/10 × 10^6^ cells. In other words, pilot experiments with other cell lines should ensure that the observed lipid concentrations with specific cell numbers and cell types are within the linear range and at least 1.5 times higher than the observed blank values. For our data presented here, many immune cells showed FFA concentrations well above 2× the blank value and were thus considered further for data analysis. Furthermore, the platform performed very robust when comparing the obtained results for a repeated analysis of 5 × 10^6^ HL60 cells, resulting in CV values < 15% relative standard deviation. As FFA showed stable results with respect to standard deviation during our study and we observed concentrations well within the established linear range, we took the obtained data along for analysis. In the case of DCER, we decided to exclude the obtained data from further evaluations as the observed analytical performance was poor for this lipid class. It is important to note that as for any lipidomics or metabolomics screening platform, specific validation steps should be undertaken when further investigating biological and phenotypic relations.

**Table 1:** Linearity and repeatability of IPA extraction methodology. Human lymphoma cell line HL60 was cultured in RPMI 10% FCS and 1% PS. Linearity was assessed by calculating the correlation coefficient (Corr) using different numbers of cells (1.25, 2.5, 5.0, and 10.0 × 10^6^). For repeatability, coefficient of variation (%CV) was used as a parameter, and was evaluated on four replicates (5.0 × 10^6^). See also ESM S6

Cell # × 10^6^	CE	CER	DAG	DCER	FFA	HCER	LCER	LPC	LPE	PC	PE	SM	TAG
1.25	7.6	1.1	3.9	0.6	29.2	1.1	3.6	0.4	0.2	214.2	121.7	89.7	11.3
2.5	13.4	2.1	6.7	0.8	27.1	2.1	6.9	1.0	0.5	427.5	245.9	158.2	20.2
5	23.1	4.3	13.7	1.1	25.4	4.1	14.1	1.9	0.9	842.2	457.3	282.1	40.9
10	32.9	7.3	23.5	0.7	65.8	7.2	24.1	7.0	1.7	1401.5	413.7	417.0	72.1
25	120.4	8.8	90.0	3.8	91.9	22.5	49.5	13.8	3.8	3034.0	1385.2	681.8	114.1
50	243.5	15.3	ND	ND	135.2	43.9	ND	25.9	8.3	5617.7	2394.2	1262.3	217.5
Blank	0.5	0.05	ND	3,1	22.9	ND	ND	ND	ND	ND	ND	3.3	0.8
Coeff. corr	*1.00*	*0.93*	*0.98*	*0.87*	*0.93*^*a*^	*1.00*	*0.99*	*0.99*	*1.00*	*1.00*	*0.99*	*0.99*	*0.99*
%CV	*2.73*	*4.40*	*8.64*	*12.20*	*22.57*	*6.43*	*8.78*	*3.77*	*3.05*	*6.60*	*12.45*	*5.50*	*3.96*

Data represent the mean of the concentration (nmol/10^6^ cells) for the 13 lipid classes

*ND* not determined

^a^Linearity ranged from 5 until 50 × 10^6^ cells

### Lipid profiling at the unstimulated state

**Fig. 2:**
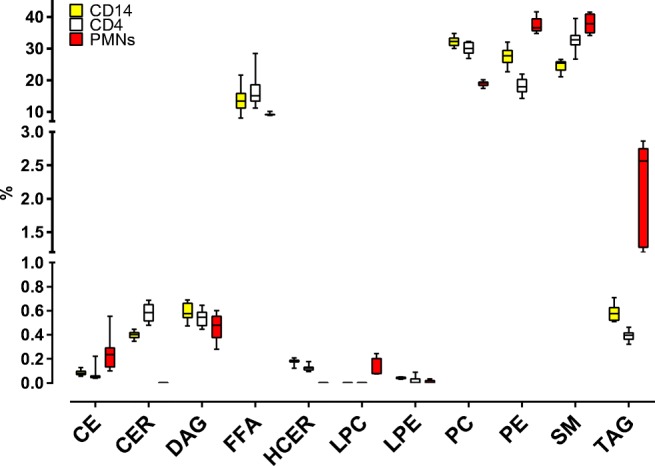
Lipid composition comparison of CD4+, CD14+, and PMNs. Cells from three donors were analyzed, and three technical replicates (5 × 10^6^ cells per replicate) were measured. Data represent the mean ± SD of the lipid class fraction percentage

We analyzed the lipid profiles of CD14+, CD4+, and PMN. After lipid extraction with IPA, three replicates from three donors were measured resulting in nine replicates per cell population. Our first aim was to draw a comparison between the lipid profiles of the different immune cell populations at the unstimulated state. We used the Kruskal-Wallis test to compare among lipid classes in each population (ESM S7) and found that, except for LPE, most of the lipid classes showed significant differences specific to the immune cell type. Figure 2 depicts the behavior for the different lipid classes among the three populations. The CD4+ and CD14+ population were quite similar to each other. This finding was also supported by a Mann-Whitney test (ESM S8) in which DAG, and FFA, showed no significant differences between these two populations. PMN, on the other hand, proved to be divergent (Fig. 2 and ESM S8). In particular, PMN showed the highest fraction of TAG lipids and they were the only population with measurable LPC class lipids. CD4+ cells displayed a trend towards an increased CER fraction, showing a high percentage of the lipidome consisting of SM, CER, and DCER lipids. Moreover, CD4+ together with CD14+ cells showed higher levels of FFA and PC class lipids.

### Lipid profiling under stimulation

**Fig. 3:**
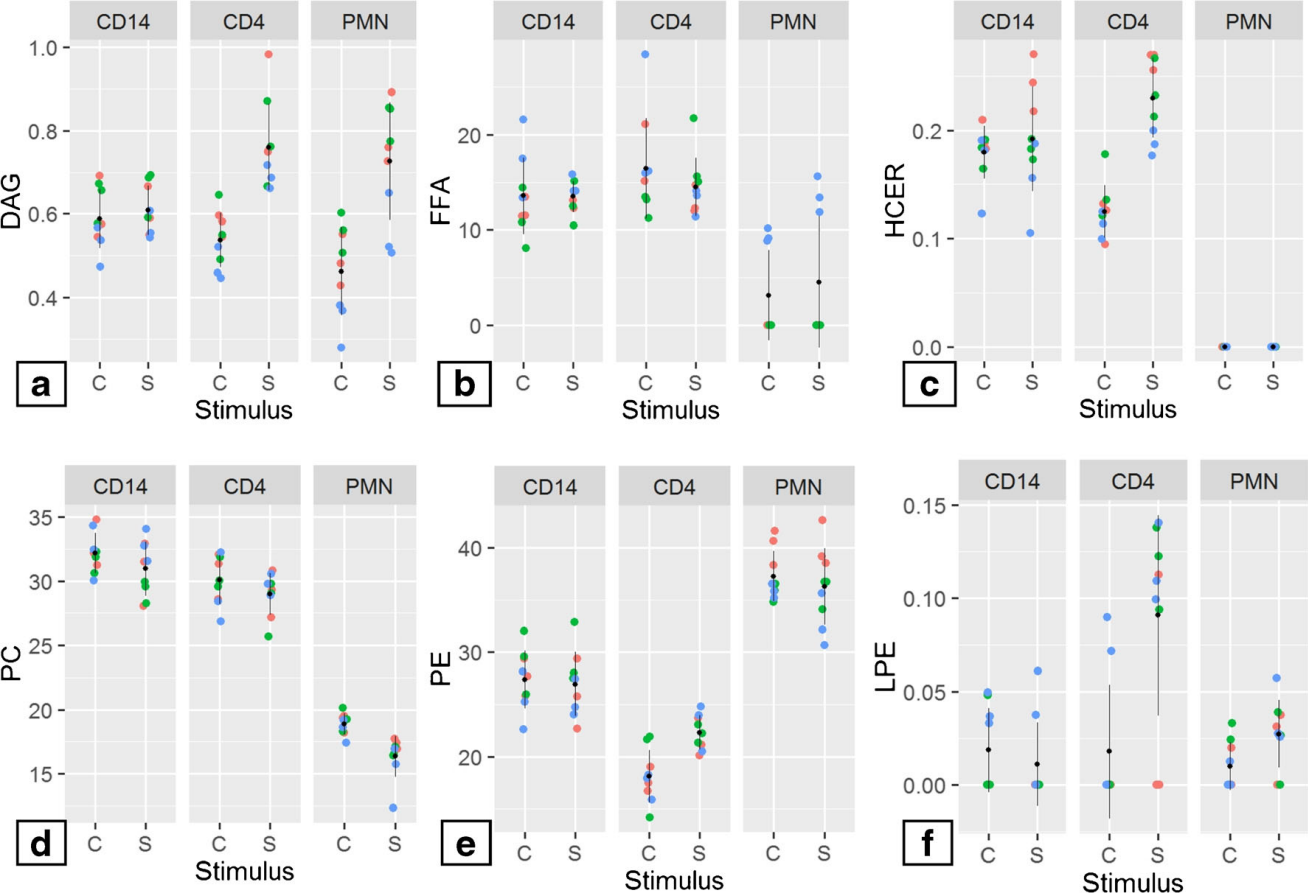
Stimulation of CD4+, CD14+, and PMNs induces significant changes in DAG, FFA, HCER, PC, PE, and LPE. Cells from three donors were isolated and stimulated with calcium ionophore (CD14 and PMNs) or CD3/CD28 (CD4); three replicates (5 × 10^6^ cells per replicate) were measured. Results from multiple Student’s *t* test showed six lipid classes mainly affected after cell stimulation: **a** DAG, **b** FFA, **c** HCER, **d** PC, **e** PE, and **f** LPE. Data show composition percentage, *p ≤ 0.05, **p ≤ 0.01, ***p ≤ 0.001, ****p ≤ 0.0001. The significance level was set at *p* < 0.05. C, control; S, stimulation; blue, donor 1; green, donor 2; red, donor 3

Next, we moved forward to explore changes in lipid metabolism after stimulation. To this end, we treated PMNs and CD14+ cells with calcium ionophore for 10 min; CD4+ cells were treated with CD3/CD28 antibodies overnight which induces activation of the cells and proliferation. We used a multiple Student’s *t* analysis to evaluate changes after stimulation within each population (ESM S9). Figure 3 shows the lipid classes presenting a major variation after stimulation. Our results show that PMN after calcium ionophore stimulation had a significant rise in the DAG and FFA fraction (Fig. 3a, b), and a reduction of the PC class (Fig. 3d). On the other hand, the CD4+ population showed major changes after antibody stimulation, in which the fraction of DAG, HCER, PE, and LPE was increased compared to the unstimulated state (Fig. 3a, c, e, f). Interestingly, CD14+ cells showed no significant differences in the lipid profile after 10 min of ionophore stimulation.

### CD4+ lipid profile after stimulation

**Fig. 4:**
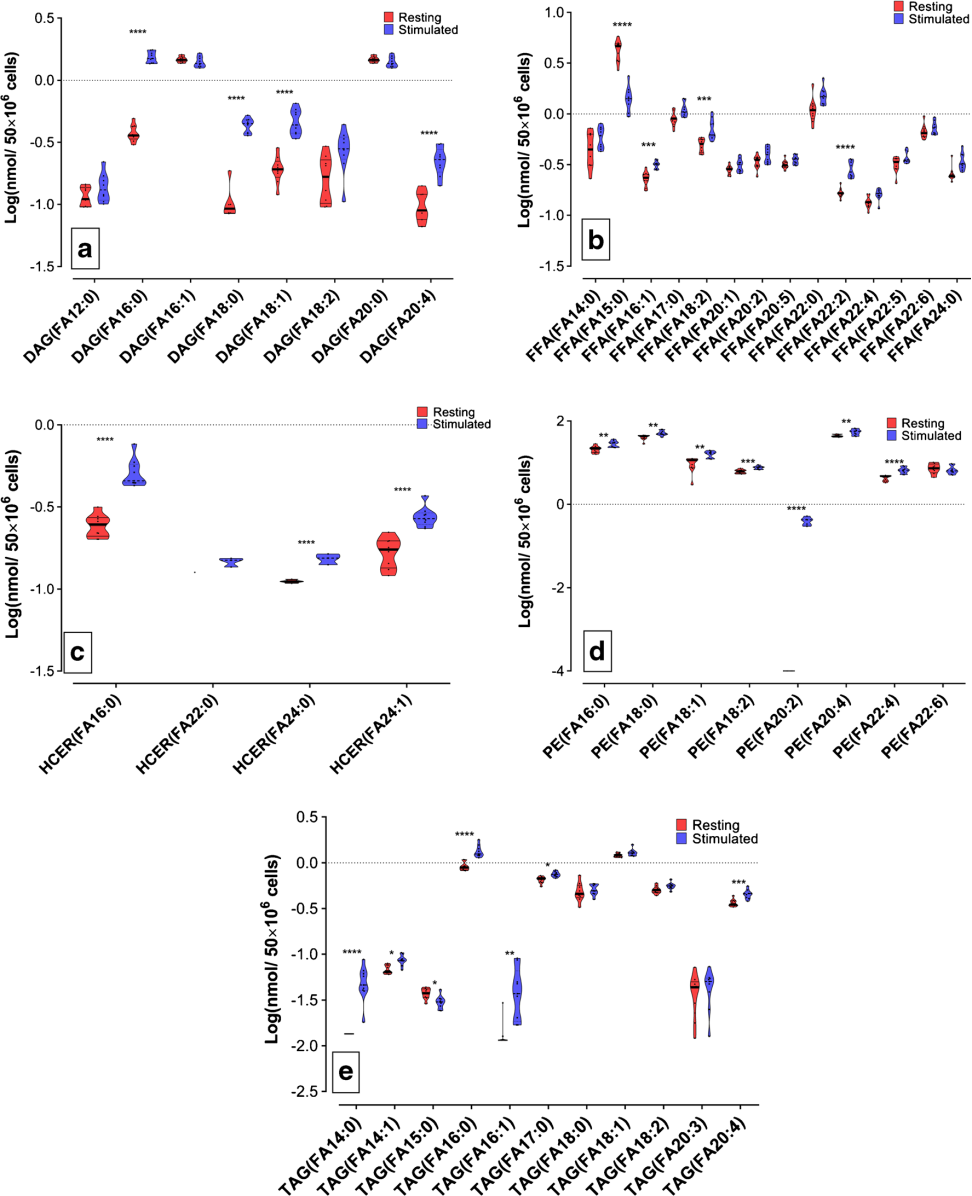
Fatty acid concentration in different lipid classes after activation of CD4 cells with CD3/CD28 antibodies. CD4+ cells from three donors were isolated and stimulated with CD3/CD28; three replicates (5 × 10^6^ cells per replicate) were measured. Lipid classes showing statistical most significant changes were selected. The FA side chain concentration was quantitatively measured. Graphics depicted species with the most significant variations, upon Log transformation. **a** DAG, **b** FFA, **c** HCER, **d** PE, and **e** TAG. *p ≤ 0.05, **p ≤ 0.01, ***p ≤ 0.001, ****p ≤ 0.0001. The significance level was set at *p* < 0.05

We could identify specific lipid classes involved in the activation of CD4+ cells and PMN after specific stimulation with anti CD3/CD28 or calcium ionophore, respectively. As FAs are known to be important precursors for several lipid mediators such as for example the eicosanoids and docosanoids [20], we specifically explored the FA concentration instead of composition for the significantly affected lipid classes. We decided to use lipid concentration because fractional lipid composition is based on the total number of measured species; hence, in those lipid classes in which only a few or one species was measured, the alterations upon stimulation were less evident. For CD4+ cells, we analyzed FA changes in the DAG, HCER, and PE lipid classes, respectively. Figure 4a, c, d shows the FAs with significant changes related to the aforementioned lipid classes. Interestingly, increased concentrations of palmitic acid (FA (16:0)) were a common feature shared by all three lipid classes. The DAG and PE classes presented important changes in the concentration of long-chain and polyunsaturated fatty acids, such as stearic (FA (18:0)), oleic (FA (18:1)), linoleic (FA (18:2)), and arachidonic acid (FA (20:4)). The HCER and PE classes displayed a significant boost in the FA concentration of long-chain FA. In order to investigate a possible source for the observed changes in the FA composition, we assessed the FA content for the FFA and TAG lipid classes (Fig. 4b, e). In particular, myrisitic acid (FA (14:0)) was increased after stimulation; at the same time FA (16:0), FA (16:1), and FA (20:4) showed increased concentration in the TAG lipid class. A common feature in FFA and TAG was an increased concentration of palmitoleic acid (FA(16:1)).

### PMN lipid profile after stimulation

**Fig. 5:**
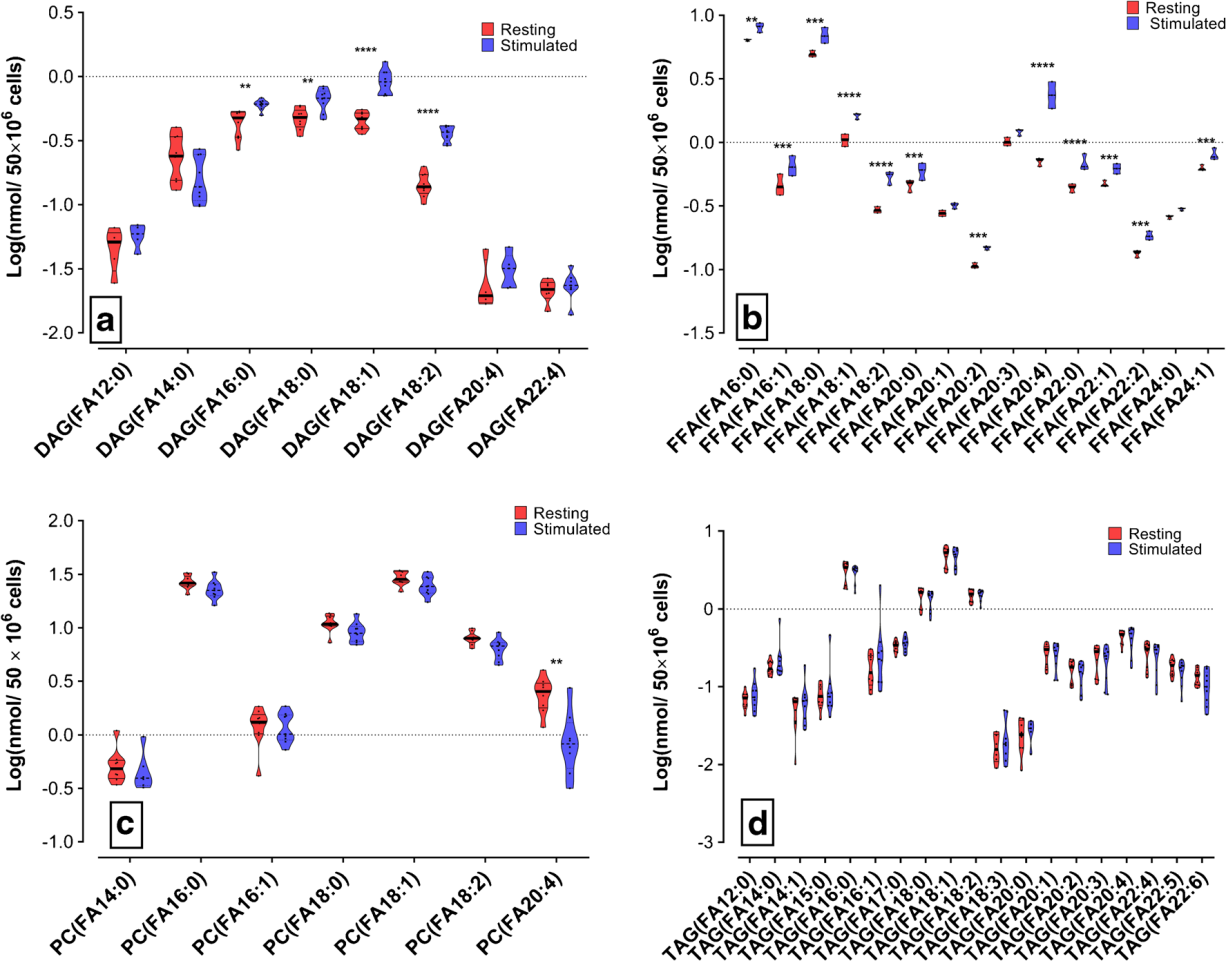
Specific fatty acids being involved during the PMN stimulation with calcium ionophore. PMN cells from three donors were isolated and stimulated with calcium ionophore; three replicates (5 × 10^6^ cells per replicate) were measured. Lipid classes showing the statistically most significant change were selected. Fatty acid side chain concentration was quantitatively measured. Graphics depicted species with the most significant variations, upon Log transformation. **a** DAG, **b** FFA, **c** PC, **d** TAG. *p ≤ 0.05. **p ≤ 0.01. ***p ≤ 0.001. ****p ≤ 0.0001. The significance level was set at *P* < 0.05

PMN showed significant differences for the DAG, FFA, and PC (Fig. 3a, b, d) lipid classes. Increased concentrations in FA (16:0), FA (18:0), FA (18:1), and FA (18:2) were common for DAG and FFA (Fig. 5a, b). Calcium ionophore stimulation also augmented the concentration of FA (20:4) and very long chain fatty acids in the FFA lipid class. However, when specifically analyzing the FA content of the PC fraction, we found that only FA (20:4) showed a significant change correlating with calcium ionophore stimulation (Fig. 5c).

## Discussion

In the present study, we applied a quantitative DMS-based lipidomics platform (Lipidyzer™) to comprehensively study the lipidome of different immune cells in unstimulated and activated states, using IPA for lipid extraction. In total, we detected #790 unique lipid species (including 26 CE, 11 CER, 7 DCER, 38 DAG, 26 FFA, 7 HCER, 5 LCER, 15 LPC, 6 LPE, 62 PC, 91 PE, 12 SM, and 484 TAG species) present in CD4+, CD14+, and PMN cell extracts (ESM S5).

We tested the linearity and repeatability of this methodology using human leukemia cells (HL60). Ten out of the thirteen evaluated lipid classes displayed a correlation coefficient above 0.95 (Table 1), showing a good linearity of the methodology. For repeatability, four biological replicates from a single cell batch were tested. Table 1 shows %CVs for the different lipid classes; with the exception of DCER and FFA, all the lipid classes had values below 15%, indicating low variability in replicates or methodology. Even though, compared to Bligh and Dyer, or MTBE, IPA is a less popular methodology, for our purpose, this lipid extraction worked properly as can be judged by good %CVs and linearity.

We profiled three different immune cell populations CD14+, CD4+, and PMN (Table 2). Today, only few reports describe the lipidomics analysis of immune cells, PBMCs [21], lymphocytes, and PMNs [22], and red blood cells (RBC) [23]. However, most studies carried out analysis solely at resting state. Just recently, a study by Leidl et al. [24] compared the lipid species in monocytes, lymphocytes, granulocytes, platelets, and RBC of healthy volunteers at resting state using flow injection ESI tandem mass spectrometry. The authors found PE, SM, and PC species to be the most prominent lipid classes found in the investigated cell types. Another study investigated the lipidome of PMN and particularly found high levels of TAG lipids in these cells [25]. In our study, we could confirm this finding, proving that particularly PE, PC, and SM lipids were prominent in the investigated immune cells. A specific characteristic of the CD4+ population was high percentages of CER, and SM lipids, pinpointing to a particular involvement of these cells in CER metabolism. Indeed, CER metabolism has been related to the activation and pro-inflammatory response in T cells [26, 27]. It is important to highlight that these cell types are very different in size and granularity (morphology); consequently, they have a different composition of cell membrane/intracellular membrane/compartments. This probably explains much of the differences we found.

**Table 2:**
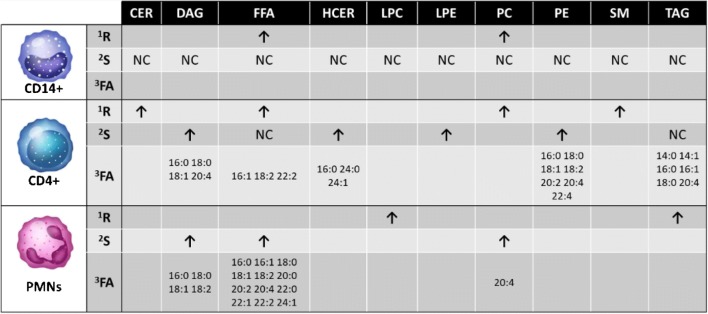
Summary. ^1^*R* significant differences in the resting state compared with the other cell populations under study. ^2^*S* significant differences after stimulation comparing unstimulated with resting state. ^3^FA with a significant change after stimulation shown for the specific lipid class. Direction of arrow shows increase in lipid composition (lipid classes) or concentration (FA content). *NC* no change

When comparing the lipidomes derived from activated immune cells with the resting state, several significant observations could be made. In the CD4+ population, notable changes in the DAG, HCER, and PE lipid classes were observed. Interestingly, DAG species with elongated FA (FA > 16) and PUFA side chains were predominant. This upregulation is a common feature after lymphocyte activation, in which DAG lipids are released mainly from membrane phospholipids [28] by the actions of phophatidylinositol and phosphatidylcholine-dependent phospholipases. Once the intracellular concentration of DAG lipids is increased, they can act as signal transducers by stimulating the enzyme protein kinase C, and downstream regulate cell differentiation and activation [29]. In our hands, mainly PE (Fig. 3d) and PC (ESM S1) were affected. However, contrary to what we expected, they did not seem to be the source of increased DAG lipids. In order to investigate another possible origin of the observed PUFA side chains, we investigated the TAG and FFA lipid classes. TAG showed increased concentration in saturated fatty acid, specifically FA (14:0) was boosted (Fig. 4e); this observation can be the result of a mechanism to control cytotoxic FFA in the cytoplasm by increasing TAG synthesis and lipid droplets as previously described [30].

With regard to the observed increase of HCER lipids under activation of CD4+ cells, the work by Hanief Sofi and co-workers showed that de novo *s*ynthesis of CER is an important feature involved in T cell proliferation and inflammatory response [27]. Their work underlined CER synthetase 6 (CerS6) to be involved in the biosynthesis of C16 CER being important in T cell biology. In turn, the here observed upregulation of HCER lipids can possibly be related to the actions of CerS6, and likely other CER synthases.

Finally, we found an upregulation of PE lipids under CD4+ stimulation. When investigating the FA composition of this lipid class, we found an unexpected increased amount of PE lipids containing arachidonic acid (FA20:4) and its elongation product adrenic acid (FA22:4). In 2004, Tomita and co-workers showed that during CD4+ cell activation arachidonic acid was unidirectionally transferred from PC to PE [31]. In the present study, we observed an increase in PE concentration (Fig. 3e), including arachidonic acid, other PUFAs, and long-chain fatty acids (Fig. 4d). In contrast with the results of Tomita, we did not see significant changes in the PC fraction (ESM S9). However, our results in PC-derived FA concentration showed a significant reduction in the PC-derived FA (20:3) (ESM S10). Our results also showed a clear PE enrichment, but the biological significance of it and its biosynthetic pathway should be subject to further studies.

Surprisingly, CD14+ cells presented no significant changes after calcium ionophore stimulation (ESM S11) even though we observed a trend towards increased concentrations of DAG, FFA, PC, and SM lipids. This observation is in correlation with a decrease in the TAG, and CER lipid classes (ESM S11).

Under calcium ionophore stimulation, PMN showed an upregulation in the DAG and FFA lipid classes. This finding is very well in line with the report by Schlager et al. [32], showing the involvement of adipose triglyceride lipase (ATL) during PMN activation. Importantly, while Schlager et al. mainly focused on the production of downstream eicosanoids and their correlation with the activity of ATL, we here provide evidence for this mechanism on the level of the DAG and FFA lipid class. Further evaluating this relation in more detail, we investigated the FA concentration of the produced DAG and FFA lipids in order to shed some light on the specificity of this process for certain lipid classes. Both lipid classes showed an accumulation of saturated C16 and C18 FA, as well as mono- and di-unsaturated C18 lipids; specifically, PUFAs were found to accumulate in the FFA fraction. Interestingly and in line with other reports [33, 34], we did find a predominant upregulation of arachidonic acid (FA20:4) levels after calcium ionophore stimulation. While the origin of this FA has been coined to be the inner membranes of PMN as well as TAG lipids [35], it was still remarkable to see that the only fatty acid being downregulated was arachidonic acid. This speaks for the fact of a highly selective action of cytosolic phospholipase on the PE lipid class.

In conclusion, the present work is a primer for the use of the Lipidyzer™ platform for cellular lipid profiling. The platform behaved overall linear and provided very robust and reproducible results. Although we did not compare efficiencies with other extraction methodologies, IPA extraction of cellular lipids proved to be linear and reproducible. This will mainly be related to limited matrix effects encountered in cellular incubations and should not be generalized towards more complicated matrices such as serum or plasma. Moreover, some pilot experiments depending on the cell line under investigation seem mandatory when evaluating other cell systems. However, using approximately 5 × 10^6^ cells, we obtained highly robust and reproducible results allowing to carry out sound statistical analysis of the data resulting in molecular observations, which align very well with other reports. Nevertheless, as for all metabolic screening platforms, specific validation experiments should be considered when moving forward into biological and phenotypic correlations. Our here-presented results show that the Lipidyzer™ platform can serve as an intriguing quantitative lipidomics platform for studying cellular lipid metabolism and generating new biological hypothesis. It will be important that the Lipidyzer™ community will evaluate and provide cell and tissue specific protocols further expanding the usefulness of this platform and allowing its validation for specific applications.

## Electronic supplementary material

ESM 1 (PDF 5.42 kb)

ESM 2 (XLSX 397 kb)
