# Supplementary material for: Lipid metabolism of leukocytes in the unstimulated and activated states
Source: Anal Bioanal Chem. 2020 Feb 14;412(10):2353–63. doi: 10.1007/s00216-020-02460-8 (PMC7118052; doi:10.1007/s00216-020-02460-8)
Supplement: Supplementary file 1 — (PDF 5.42 kb) [file 216_2020_2460_MOESM1_ESM.pdf]

Analytical and Bioanalytical Chemistry

Electronic Supplementary Material

Lipid metabolism of leukocytes in the unstimulated and activated states

Juan Carlos Alarcon-Barrera, Johannes H. von Hegedus, Hilde Brouwers,
Evelyne Steenvoorden, Andreea Ioan-Facsinay, Oleg A. Mayboroda,
Alejandro Ondo-Mendez, Martin Giera

Additional file available under 10.1007/s00216-020-02460-8.
